# Totally endoscopic mitral valve replacement: reasons for non-feasibility of repair and 5-year outcomes

**DOI:** 10.3389/fcvm.2026.1903773

**Published:** 2026-10-20

**Authors:** Friedrich Sobik, Jonas Pausch, Xiaoqin Hua, Martin Beyer, Evelina Macius, Oliver Bhadra, Yousuf Alassar, Benjamin Kloth, Lenard Conradi, Hermann Reichenspurner, Simon Pecha, Evaldas Girdauskas, Andreas Schaefer

**Affiliations:** 1 Department of Cardiac Surgery, University Heart and Vascular Center Hamburg, University Medical Center Hamburg-Eppendorf, Hamburg, Germany; 2 Department of Cardiac Surgery, University Hospital Cologne, Cologne, Germany; 3 DZHK (German Centre for Cardiovascular Research), Partner Site North, Hamburg, Germany

**Keywords:** bioprostheses, endoscopic mitral valve surgery, minimally invasive mitral valve surgery, mitral valve (MV), mitral valve replacement

## Abstract

**Objectives:**

Totally endoscopic mitral valve (MV) surgery is an established therapy for mitral regurgitation (MR) with high repair rates at experienced centers. In certain scenarios, however, repair is not feasible, necessitating chordal-sparing MV replacement. This study investigates the reasons for totally endoscopic MV replacement and reports 5-year outcomes.

**Methods:**

Between 2012 and 2022, 1,037 consecutive patients underwent totally endoscopic MV surgery. Of those, 75 patients (7.2%) underwent chordal-sparing MV replacement. Preoperative characteristics, periprocedural data, and mid-term outcomes were retrospectively analyzed and compared to a 1:1 propensity score-matched repair group.

**Results:**

After matching, baseline characteristics were balanced. Indications for MV replacement included sclerotic leaflets (28.0%), endocarditis (26.7%), MV stenosis (17.3%), failed repair attempt (17.3%), rheumatic disease (12.0%), and residual MR after transcatheter edge-to-edge repair (7%). Cross-clamp and bypass times were similar. Re-thoracotomy occurred in 14.7% vs. 6.7%, while permanent pacemaker implantation occurred solely after replacement (16.0%). Low cardiac output rates were comparable (6.7% vs. 5.5%). Transvalvular gradients at discharge were higher after replacement (*p* < 0.001). No paravalvular leakage was observed. In-hospital mortality was 4% vs. 0%. Five-year survival was 86.7% vs. 94.7%, and reintervention rates were similar (4.0% vs. 6.7%).

**Conclusion:**

Totally endoscopic MV surgery at a high-volume center is infrequently associated with MV replacement. The main reasons included sclerotic leaflet pathology, MV stenosis, and endocarditis. Despite a higher comorbidity burden and residual baseline imbalance after matching, early and mid-term outcomes were acceptable. Totally endoscopic chordal-sparing MV replacement represents a safe and clinically effective treatment option for selected patients.

## Introduction

Mitral regurgitation (MR) is the second most common valvular heart disease in Europe and increases in prevalence with age (1). Conventional mitral valve (MV) surgery (MVS) via full sternotomy, including central cannulation for cardiopulmonary bypass (CPB) and cardioplegic arrest, has long been considered the gold standard for treating MR and is supported by current guidelines (2). Over the past two decades, endoscopic MV surgery has emerged as an alternative to conventional sternotomy (3, 4), offering a complete range of MV repair (MVr) techniques that address all types of MR pathologies (5–8). Within minimally invasive mitral valve surgery, both direct-vision and totally endoscopic approaches have been described, each with distinct technical requirements and learning curves (9). Endoscopic MVS has been associated with reduced surgical trauma, decreased need for transfusions, shorter postoperative ventilation times, and shorter in-hospital stays (10–15). However, rates of successful MVr are strongly correlated with the experience of centers and surgeons, resulting in uneven region-specific distributions of MV replacement (MVR) rates (16). This is clinically relevant, as several studies have demonstrated the superiority of MVr over replacement in selected patient populations, including those with reduced left ventricular ejection fraction (LVEF) and elderly patients (17, 18). Conversely, in patients with severe ischemic secondary MR, the randomized Cardiothoracic Surgical Trials Network (CTSN) trial demonstrated significantly lower recurrence of MR after chordal-sparing replacement compared with repair, with comparable survival and left ventricular reverse remodeling at two years (19, 20)—one of the strongest evidence-based exceptions to a repair-first strategy. Nonetheless, there are certain patient and anatomical circumstances in which MVr is not feasible and MVR is performed. At our center, an MVr rate of over 90% has been achieved in recent years, largely through a totally endoscopic minimally invasive cardiac surgery (MICS). The aim of this study was to identify reasons for MVR during MICS MV procedures and to report mid-term outcomes of these procedures up to 5 years.

## Patients and methods

This retrospective single-center study was conducted according to the ethical standards of the Declaration of Helsinki (1964). Given the retrospective, anonymized study design, ethical approval was not required in accordance with local legislation and institutional requirements, and the requirement for written informed patient consent was waived in accordance with national legislation and institutional requirements.

All consecutive patients (*n* = 1037) receiving endoscopic MICS MVS between 2012 and 2022 were retrospectively included in the registry. The indication for surgery followed current guidelines of the European Society of Cardiology/European Association for Cardio-Thoracic Surgery (ESC/EACTS) for managing valvular heart disease (2). Furthermore, the decision for endoscopic MVS was based on local standards of care and interdisciplinary Heart Team discussions. Of those 1,037 patients, 75 underwent MVR (study group). To further elucidate outcomes for these patients, matching was conducted with the remaining 962 patients to create a control group of 75 patients who underwent MICS MVr. The primary endpoints for this study were mortality and mitral valve reintervention.

### Surgical setup

All procedures were performed under general anesthesia by a dedicated team of cardiac surgeons, anesthesiologists, nurses, and perfusionists according to institutional standards. Briefly, endoscopic MVS was performed via a right anterolateral or transaxillary mini-incision under 3D endoscopic guidance using a non-rib-spreading soft tissue retractor. CPB was established via percutaneous or open cannulation of femoral or axillary arteries and femoral veins. A transthoracic aortic clamp and antegrade del Nido cardioplegia were utilized to achieve cardioplegic arrest. Exposure of the MV was obtained through direct left atrial access, followed by a standardized intraoperative examination to determine the etiology of MR and define the surgical strategy. MVR and repair, as well as concomitant surgical procedures (atrial fibrillation ablation, left atrial appendage closure, tricuspid valve surgery), were performed according to local standards of care. The preservation of the subvalvular apparatus during MVR was undertaken whenever anatomically feasible, at the discretion of the attending surgeon; preservation was not attempted in the presence of significant annular calcification or a small, restrictive annulus. Typically, only the posterior mitral leaflet (PML) and its chordal attachments were preserved, while the anterior leaflet was resected; the preserved posterior leaflet was incorporated directly into the valve placement sutures, with excision of the central leaflet portion if excessive bulk hindered adequate prosthesis seating. In select cases where anterior leaflet preservation was chosen, a triangular resection of the leaflet base was performed, and the remaining tissue was similarly incorporated into the valve placement sutures. The strategy was tailored to valve pathology and intraoperative anatomy rather than applied uniformly. During the procedure, transesophageal echocardiography (TEE) was used to confirm satisfactory surgical results.

### Endpoint definitions

Outcome definitions were as follows: low cardiac output syndrome was defined as the need for extracorporeal membrane oxygenation, Impella support, or prolonged catecholamine requirements exceeding 24 h. Acute kidney injury (AKI) was classified according to the Kidney Disease: Improving Global Outcomes (KDIGO) criteria, with only stage 3 or higher considered clinically relevant AKI for this analysis. Postoperative dialysis was defined as a new-onset requirement for dialysis due to impaired renal function with hypervolemia and/or hyperkalemia. Right heart failure was defined as the need for inotropic or mechanical right ventricular support. Re-thoracotomy for bleeding was defined by prolonged chest tube output despite optimized coagulation status, total drainage exceeding 1,200 mL, hemothorax, or a significant hemoglobin drop, in line with the bleeding severity framework proposed by the Mitral Valve Academic Research Consortium (MVARC) (21). Reintervention was defined as any repeat mitral valve-related procedure, whether surgical or transcatheter. In-hospital and 30-day mortality were recorded as standard endpoints. Vital status beyond the index hospitalization was ascertained through standardized institutional follow-up procedures, providing complete vital status information for the entire matched cohort.

### Statistical analysis

Baseline, perioperative, and follow-up data were retrospectively collected in an anonymized, standardized database. Continuous variables are shown as mean ± standard deviation and compared using the Mann–Whitney U-test. Binary variables are shown as counts (frequencies) and compared using the *χ*^2^ test. Event rates and survival curves were calculated using the Kaplan–Meier estimator. Groups were compared using the log-rank test.

For all 1,037 patients, propensity scores were calculated using a logistic regression model that included the covariates: age, sex, body mass index (BMI), history of atrial fibrillation, and arterial hypertension. Matching was conducted with a caliper of 0.2, resulting in 75 matched pairs. Each patient in the study group was matched to a patient in the control group based on their propensity scores.

The matched pairs were then compared to analyze differences in baseline characteristics and short- and mid-term outcomes. To account for multiple comparisons across all baseline, procedural, and outcome variables reported in Tables 1–5, *p*-values were adjusted using the Benjamini–Hochberg procedure, treating all comparisons across the manuscript as a single family of tests. Adjusted *p*-values below 0.05 were considered statistically significant. All analyses were performed using R statistical software version 4.0.3 (R Foundation for Statistical Computing, Vienna, Austria).

**Table 1:** Preoperative patient characteristics.

Variable	MV replacement (*n* = 75)	MV repair (*n* = 75)	CI for difference of means (95%)	Adjusted *p*-value
Male, *n* (%)	33 (44.0)	33 (44.0)		1.0
Age, years	64 ± 10.8	64 ± 11.1	−3.13 to 3.95	0.82
BMI, kg/m^2^	25.7 ± 5.0	25.5 ± 0.4	−1.77 to 1.31	0.8
STS PROM, %	1.96 ± 1.4	0.66 ± 0.9	−1.63 to −0.98	<0.001
NYHA III, *n* (%)	32 (47.1)	25 (33.8)		0.45
NYHA IV, *n* (%)	8 (11.8)	9 (12.3)		1.0
Arterial hypertension, *n* (%)	40 (53.3)	51 (68.0)		0.46
Diabetes, *n* (%)	8 (10.7)	3 (4.0)		0.58
Extracardiac arteriopathy, *n* (%)	3 (4.1)	1 (1.3)		0.64
COPD, *n* (%)	3 (4.0)	2 (2.7)		1.0
Atrial fibrillation, *n* (%)	36 (48)	33 (44)		1.0
Prior stroke, *n* (%)	9 (12.0)	2 (2.7)		0.42
CAD, *n* (%)	18 (24.0)	13 (17.3)		0.87
Endocarditis, *n* (%)	20 (26.7)	3 (4.0)		0.005
Prior myocardial infarction, *n* (%)	2 (2.7)	4 (5.3)		0.92
Dialysis, *n* (%)	5 (6.7)	0 (0.0)		0.42
Permanent pacemaker, *n* (%)	2 (2.7)	3 (4.0)		1.0
Prior Sternotomy, *n* (%)	2 (2.7)	0 (0.0)		0.9
Prior PCI, *n* (%)	5 (6.7)	4 (5.3)		1.0
Prior TEER, *n* (%)	5 (6.7)	0 (0.0)		0.42
NT-proBNP, ng/L	2,763 ± 4,126	1,473 ± 1,776	−2,567 to −13.64	0.12
Hemoglobin, g/dL	12.0 ± 2.3	13.6 ± 1.6	0.96 to 2.26	<0.001
Creatinine, mg/dL	1.35 ± 1.37	0.95 ± 0.2	−0.72 to −0.08	0.06

MV, mitral valve; CI, confidence interval; BMI, body mass index; STS PROM, Society of Thoracic Surgeons Predicted Risk of Mortality; NYHA, New York Heart Association; COPD, chronic obstructive pulmonary disease; CAD, coronary artery disease; PCI, percutaneous coronary intervention; TEER, transcatheter edge-to-edge repair; NT-proBNP, N-terminal prohormone of brain natriuretic peptide.

**Table 2:** Preoperative echocardiography.

Variable	MV replacement (*n* = 75)	MV repair (*n* = 75)	CI for difference of means (95%)	Adjusted *p*-value
LVEF, %	58.2 ± 8.5	56.8 ± 10.3	−1.62 to 4.42	0.51
Severe MV regurgitation, *n* (%)	55 (74.3)	70 (93.3)		0.03
PML prolapse, *n* (%)	23 (33.8)	39 (52.7)		0.42
AML prolapse, *n* (%)	17 (25.0)	8 (10.8)		0.42
PML, restriction, *n* (%)	14 (20.6)	10 (13.5)		0.59
AML restriction, *n* (%)	3 (4.5)	3 (4.1)		1.0
AML flail, *n* (%)	3 (4.4)	0 (0.0)		0.37
PML flail, *n* (%)	9 (13.2)	12 (16.2)		0.92
Degenerative pathology, n (%)	48 (68.6)	55 (73.3)		0.88
Functional pathology, *n* (%)	9 (12.9)	15 (20.0)		0.76
Other pathology, *n* (%)	13 (18.6)	5 (6.7)		0.3
Rheumatic valvular disease, *n* (%)	9 (12.0)	0 (0.0)		0.02
Moderate to Severe MV stenosis, n (%)	13 (17.3)	0 (0.0)		<0.001
Vena contracta, mm	7.6 ± 2.7	8.8 ± 3.5	−1.78 to 4.26	0.56
MV Pmax, mmHg	13.49 ± 7.1	7.57 ± 3.5	−8.29 to −3.56	<0.001
MV Pmean, mmHg	5.45 ± 3.3	2.5 ± 1.1	−3.98 to −1.91	<0.001
sPAP, mmHg	37.7 ± 12.8	36.1 ± 13.6	−7.02 to 3.95	0.64
LVEDD, mm	53.9 ± 8.1	55.2 ± 7.6	−1.73 to 4.51	0.54
Severe TV regurgitation, n (%)	1 (1.4)	1 (1.4)		1.0
TAPSE, mm	22.7 ± 5.1	23.1 ± 4.7	−1.22 to 2.19	0.64

MV, mitral valve; CI, confidence interval; LVEF, left ventricular ejection fraction; PML, posterior mitral leaflet; AML, anterior mitral leaflet; sPAP, systolic pulmonary artery pressure; LVEDD, left ventricular end-diastolic diameter; TV, tricuspid valve; TAPSE, tricuspid annular plane systolic excursion.

**Table 3:** Periprocedural characteristics.

Variable	MV replacement (*n* = 75)	MV repair (*n* = 75)	CI for difference of means (95%)	Adjusted *p*-value
Emergency procedure, *n* (%)	1 (1.3)	0 (0.0)		1.0
Conversion to sternotomy, *n* (%)	7 (9.3)	1 (2.7)		0.53
Concomitant TV repair, *n* (%)	1 (1.3)	8 (10.7)		0.23
Left atrial appendage occlusion, *n* (%)	25 (33.3)	20 (26.7)		0.84
Atrial ablation, *n* (%)	17 (22.7)	16 (21.3)		1.0
Cross-clamp time, min	113.4 ± 49.6	100.5 ± 31.8	−26.37 to 0.68	0.15
CPB time, min	182.7 ± 72.1	170.8 ± 52	−32.2 to 8.41	0.48
Procedure time, min	253.9 ± 91.4	263.0 ± 84.3	−19.25 to 37.49	0.62
Ventilation time, h	10.3 ± 13.5	6.5 ± 4.7	−7.62 to −0.08	0.12
ECMO support, *n* (%)	1 (1.3)	0 (0.0)		1.0
RCx lesion, *n* (%)	1 (1.3)	0 (0.0)		1.0
Re-thoracotomy, *n* (%)	11 (14.7)	5 (6.7)		0.45
Perioperative need of dialysis, *n* (%)	6 (8.1)	1 (1.3)		0.5
Right heart failure, *n* (%)	0 (0.0)	1 (1.3)		1.0
Low-output, *n* (%)	5 (6.8)	4 (5.5)		1.0
Creatinine (peak), mg/dL	1.8 ± 1.5	1.14 ± 0.2	−1.06 to −0.24	0.01

MV, mitral valve; CI, confidence interval; TV, tricuspid valve; CPB, cardiopulmonary bypass; ECMO, extracorporeal membrane oxygenation; RCx, left circumflex coronary artery.

**Table 4:** 30-day outcomes.

Variable	MV Replacement (*n* = 75)	MV Repair (*n* = 75)	CI for difference of means (95%)	Adjusted *p*-value
Hospital stay, days	12.1 ± 8.4	8.4 ± 3.6	−5.73 to −1.57	0.004
In-hospital mortality, *n* (%)	3 (4.0)	0 (0.0)	−1.54 to 11.1	0.54
30-day mortality, *n* (%)	4 (5.3)	0 (0.0)	−0.52 to 12.93	0.043
Atrial fibrillation at discharge, *n* (%)	21 (28.4)	18 (24)	−9.67 to 18.24	0.9
Sinus rhythm at discharge, *n* (%)	46 (62.2)	56 (74.7)	−27.47 to 1.59	0.52
PPI, *n* (%)	10 (13.3)	0 (0.0)	5.66 to 22.83	0.02
LVEF at discharge, %	48.3 ± 12	50.6 ± 10.7	−5.94 to 1.34	0.5
MV Pmax, mmHg	13.6 ± 4.9	9.4 ± 4.1	−5.76 to −2.66	<0.001
MV Pmean, mmHg	5.5 ± 2.1	3.5 ± 1.3	−2.61 to −1.44	<0.001
sPAP, mmHg	28.62 ± 11.8	23.02 ± 10.3	−10.8 to −0.33	0.15
LVEDD, mm	49.29 ± 7.7	50.48 ± 8.2	−2.35 to 4.72	0.62
TAPSE, mm	13.0 ± 3.3	15.7 ± 4.3	1.21 to 4.17	0.003
Moderate MV regurgitation, *n* (%)	0 (0.0)	2 (2.7)	−9.21 to 2.58	0.88
Severe MV regurgitation, *n* (%)	1 (1.4)	0 (0.0)	−3.66 to 7.17	0.88
Severe TV regurgitation, *n* (%)	2 (2.7)	0 (0.0)	−2.58 to 9.21	0.74

MV, mitral valve; CI, confidence interval; PPI, permanent pacemaker implantation; LVEF, left ventricular ejection fraction; sPAP, systolic pulmonary artery pressure; LVEDD, left ventricular end-diastolic diameter; TAPSE, tricuspid annular plane systolic excursion.

**Table 5:** Mid-term outcomes.

Variable	MV replacement (*n* = 75)	MV repair (*n* = 75)	CI for difference of means (95%)	Adjusted *p*-value
Reoperation, *n* (%)	3 (4.0)	5 (6.7)	−11.1 to 5.39	0.36^a^
Overall mortality, *n* (%)	10 (13.3)	4 (5.3)	−1.63 to 18.04	0.27^a^
Follow-up duration, (SD), months, mean	42 (32.0)	61.7 (37.5)	8.49 to 30.98	0.004

a Log-rank test (Kaplan–Meier analysis).

As a sensitivity analysis, inverse probability of treatment weighting (IPTW, average treatment effect on the treated) was additionally performed on the full institutional cohort of mitral valve repair-eligible and replacement patients from the study period (*N* = 1037), using a propensity model that included demographic factors, surgical risk [Society of Thoracic Surgeons (STS) predicted risk of mortality score (STS PROM)], anatomical pathology, and heart failure parameters [preoperative LVEF, New York Heart Association (NYHA) class, tricuspid annular plane systolic excursion (TAPSE)]. Covariate balance was assessed using standardized mean differences (SMD) and compared with the primary 1:1 matched cohort.

## Results

### Baseline characteristics

Detailed baseline demographic characteristics are listed in Table 1. After propensity score matching for sex, age, BMI, arterial hypertension, and atrial fibrillation, baseline characteristics were largely similar between groups. However, patients undergoing MVR (study group; group 1) had a significantly higher predicted surgical risk, as indicated by the STS PROM (study group: 1.96 ± 1.4 vs. control group, group 2: 0.66 ± 0.9%, *p* < 0.001). Patients in the study group presented with significantly lower serum hemoglobin levels (12.0 ± 2.3 vs. 13.6 ± 1.6 g/dL, *p* < 0.001), and higher serum creatinine (1.35 ± 1.37 vs. 0.95 ± 0.2 mg/dL, *p* = 0.06) and NT-proBNP levels (2,763 ± 4,126 vs. 1,473 ± 1,776 pg/mL, *p* = 0.12).

Preoperative echocardiography showed higher transmitral pressure gradients (Table 2) in the study group (peak gradient: 13.5 ± 7.1 vs. 7.6 ± 3.5 mmHg, *p* < 0.001; mean gradient: 5.45 ± 3.3 vs. 2.5 ± 1.1 mmHg, *p* < 0.001). LVEF (58.2 ± 8.5 vs. 56.8 ± 10.3%, *p* = 0.51) and right ventricular function (TAPSE: 22.7 ± 5.1 vs. 23.1 ± 4.7 mm, *p* = 0.64) were similarly preserved in both groups. Furthermore, the prevalence of severe tricuspid valve regurgitation was comparable (*p* = 0.74).

### Intraoperative characteristics and reasons for MV replacement

All procedures were performed via right anterolateral/transaxillary mini-thoracotomy. Conversion to sternotomy was necessary in 9.3% of patients [*n* = 7, reasons: systemic anterior motion (SAM) phenomenon (*n* = 1), limited intraoperative visualization due to severe calcification (*n* = 1), venous congestion (*n* = 1), pulmonary adhesions (*n* = 2), and bleeding (*n* = 2)] in the MV replacement group vs. 2.7% (*n* = 2, reasons: bleeding, pulmonary adhesions) within the MVr group (*p* = 0.42). Cross-clamp, bypass, and total procedure times were similar between groups, as shown in Table 3. The anatomically and technically related conversions (SAM phenomenon, calcification-related visualization impairment, venous congestion, pulmonary adhesions; *n* = 5) were generally recognized and managed early in the procedure, whereas the bleeding-related conversions (*n* = 2) were emergent. Conversion to sternotomy was not associated with increased mortality overall, with six of the seven converted patients surviving without further sequelae.

Main indications for MVR (Figure 1) were sclerotic leaflet degeneration (*n* = 21, 28%), including rheumatic disease (*n* = 9, 12%) and/or moderate to severe MV stenosis (*n* = 13, 17.3%), acute infective endocarditis (*n* = 20, 26.7%), failed intraoperative MV repair (*n* = 13, 17.3%), or prior failed transcatheter edge-to-edge repair (TEER) (*n* = 5, 6.7%). Baseline characteristics stratified by indication for MVR, including age, sex, STS PROM, preoperative LVEF, in-hospital mortality, and reoperation rate, are provided in Supplementary Table S1.

**Figure 1:**
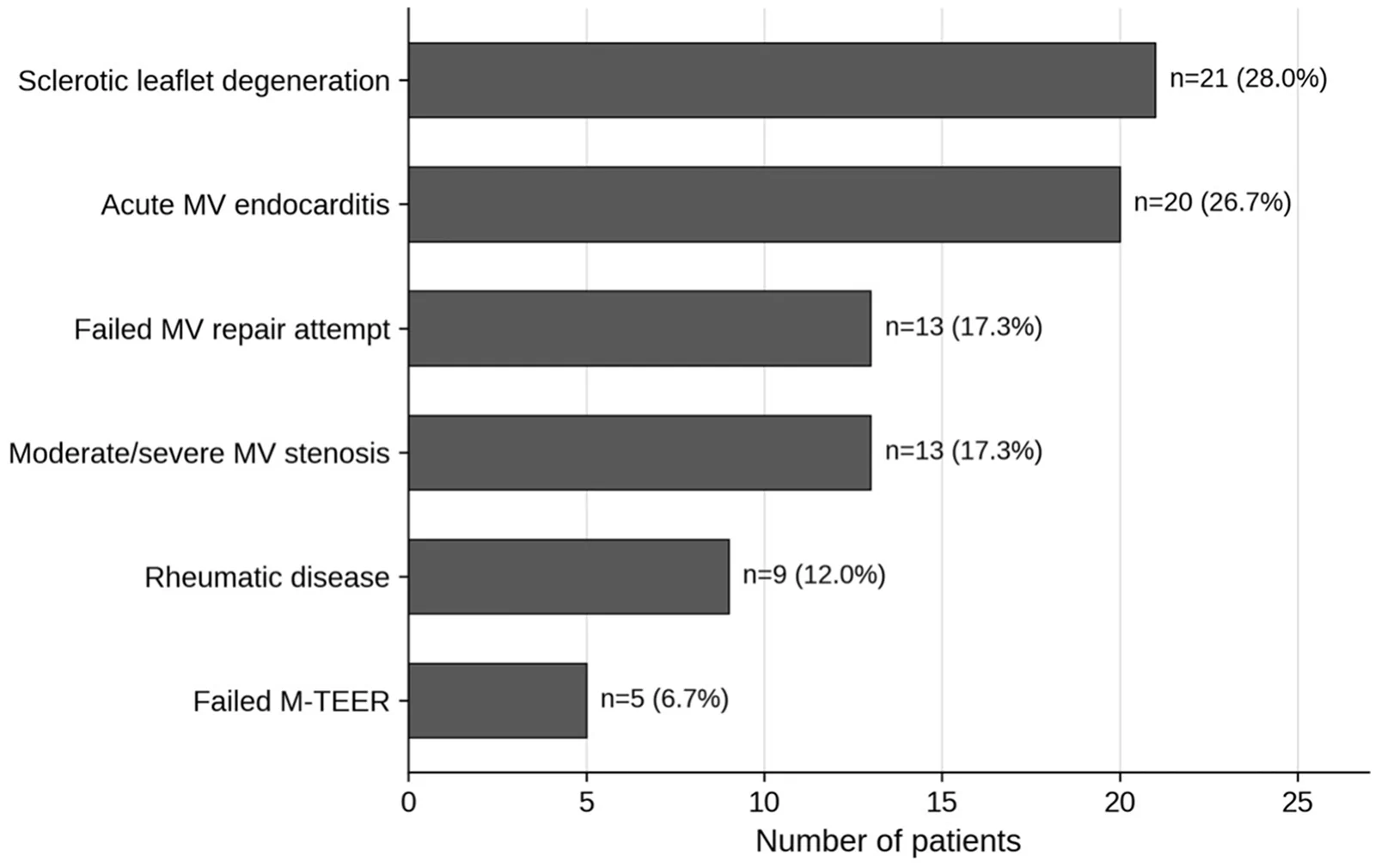
Reasons for non-feasibility of MV repair. MV, mitral valve; TEER, transcatheter edge-to-edge repair.

Bioprosthetic valves were used in 97.3% of replacement procedures. The most frequently implanted prosthesis size was 29 mm (*n* = 34, 45.3%), followed by 31 mm (*n* = 25, 33.3%), 27 mm (*n* = 9, 12.0%), 33 mm (*n* = 6, 8.0%), and 25 mm (*n* = 1, 1.3%) (Figure 2).

**Figure 2:**
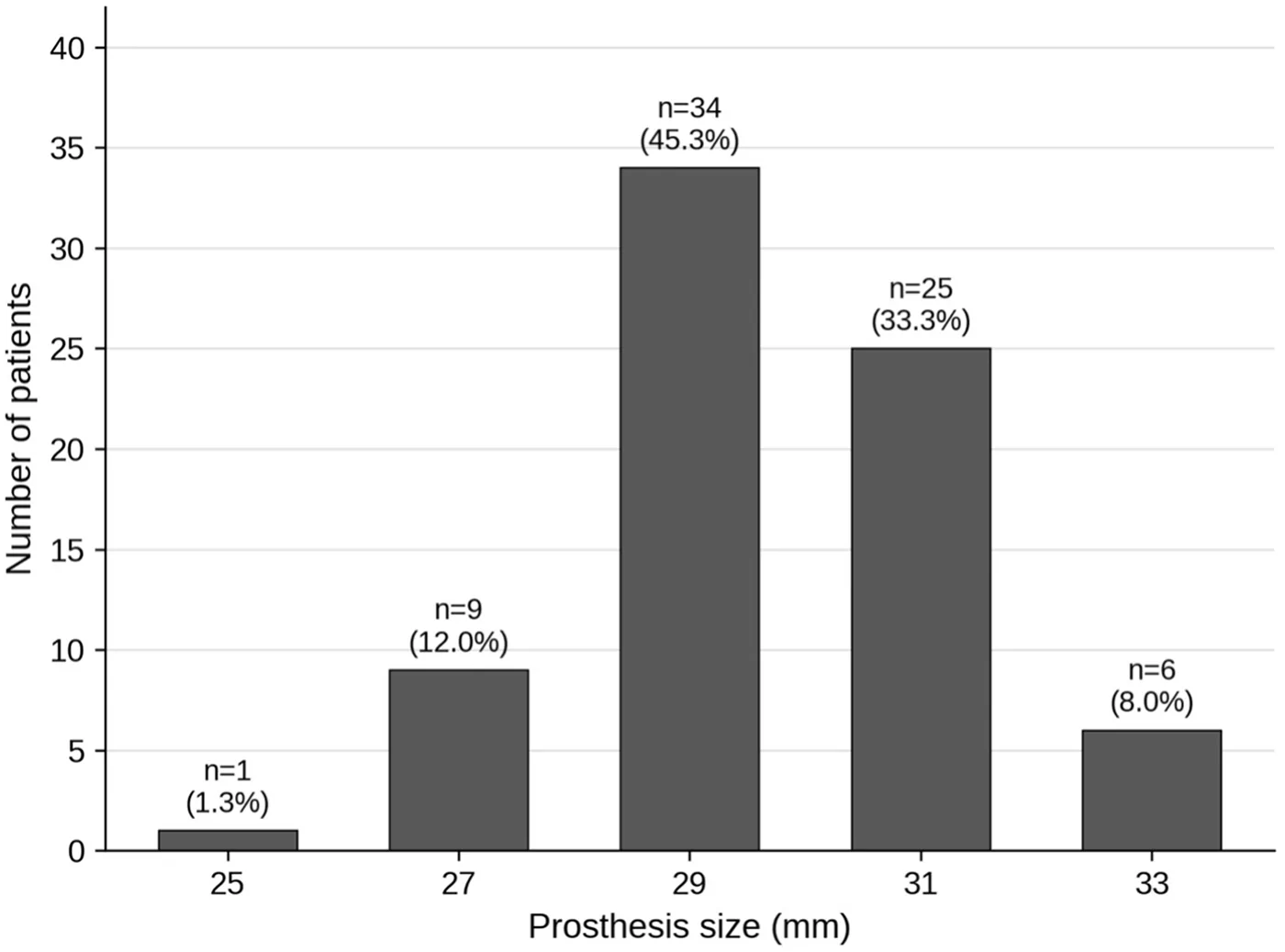
Valve sizes of implanted prostheses during totally endoscopic mitral valve replacement. *n* = 75; biological mitral valve replacement: 73/75; mechanical mitral valve replacement: 2/75. Prosthesis sizes: 25 mm (*n* = 1, 1.3%), 27 mm (*n* = 9, 12.0%), 29 mm (*n* = 34, 45.3%), 31 mm (*n* = 25, 33.3%), 33 mm (*n* = 6, 8.0%).

### Perioperative outcomes and 30-day survival

Perioperative complications, including re-thoracotomy for bleeding, acute kidney injury, and low cardiac output, were similar between groups (Table 3). As pre-specified in the Patients and methods section, re-thoracotomy was defined according to MVARC bleeding criteria; no re-thoracotomies for other indications occurred in either group. Patients undergoing MVR had a longer in-hospital stay (12.1 ± 8.4 vs. 8.4 ± 3.6 days, *p* = 0.004) and a higher rate of permanent pacemaker implantation (13.3% vs. 0%, *p* = 0.02). In-hospital mortality was 4% (3/75) in the MVR group, including patients with infective endocarditis, and 0% in the MVr group (*p* = 0.54). Thirty-day mortality, incorporating registry-confirmed vital status beyond discharge, was 5.3% (4/75) in the MVR group vs. 0% (0/75) in the MVr group; Kaplan–Meier estimated 30-day survival was 94.7% vs. 100.0%, respectively (log-rank *p* = 0.043, Figure 3).

**Figure 3:**
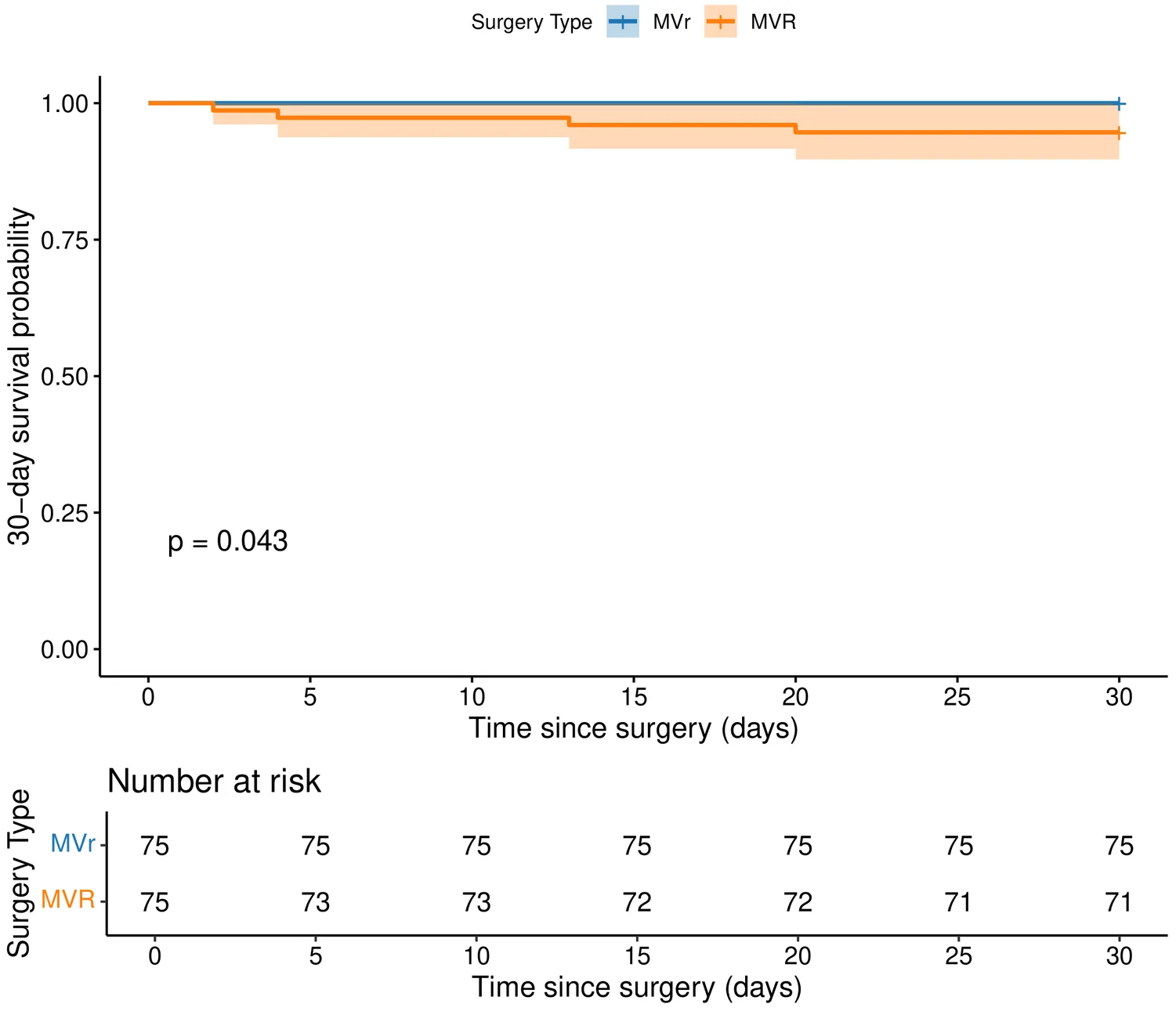
Kaplan–Meier survival curve for 30 days. MVr, mitral valve repair; MVR mitral valve replacement.

Postoperative echocardiography showed higher transmitral pressure gradients after MVR (peak gradient: 13.6 ± 4.9 vs. 9.4 ± 4.1 mmHg, *p* < 0.001; mean gradient: 5.5 ± 2.1 vs. 3.5 ± 1.3 mmHg, *p* < 0.001). Postoperative right ventricular function was lower in the MVR group (TAPSE: 13.0 ± 3.3 vs. 15.7 ± 4.3 mm, *p* = 0.003).

No differences regarding postoperative rates of residual moderate or severe MR, tricuspid regurgitation, or atrial fibrillation were seen at discharge (Table 4).

### Follow-up

Median follow-up was 46 months (IQR 21–77; Figures 4, 5), and the follow-up duration was longer in the MVr group (42.0 ± 32.0 vs. 61.7 ± 37.5 months, *p* = 0.004). Mitral reintervention rates were low and similar between groups (*n* = 3, 4.0% vs. *n* = 5, 6.7%, *p* = 0.36) (Table 5, Figure 5).

**Figure 4:**
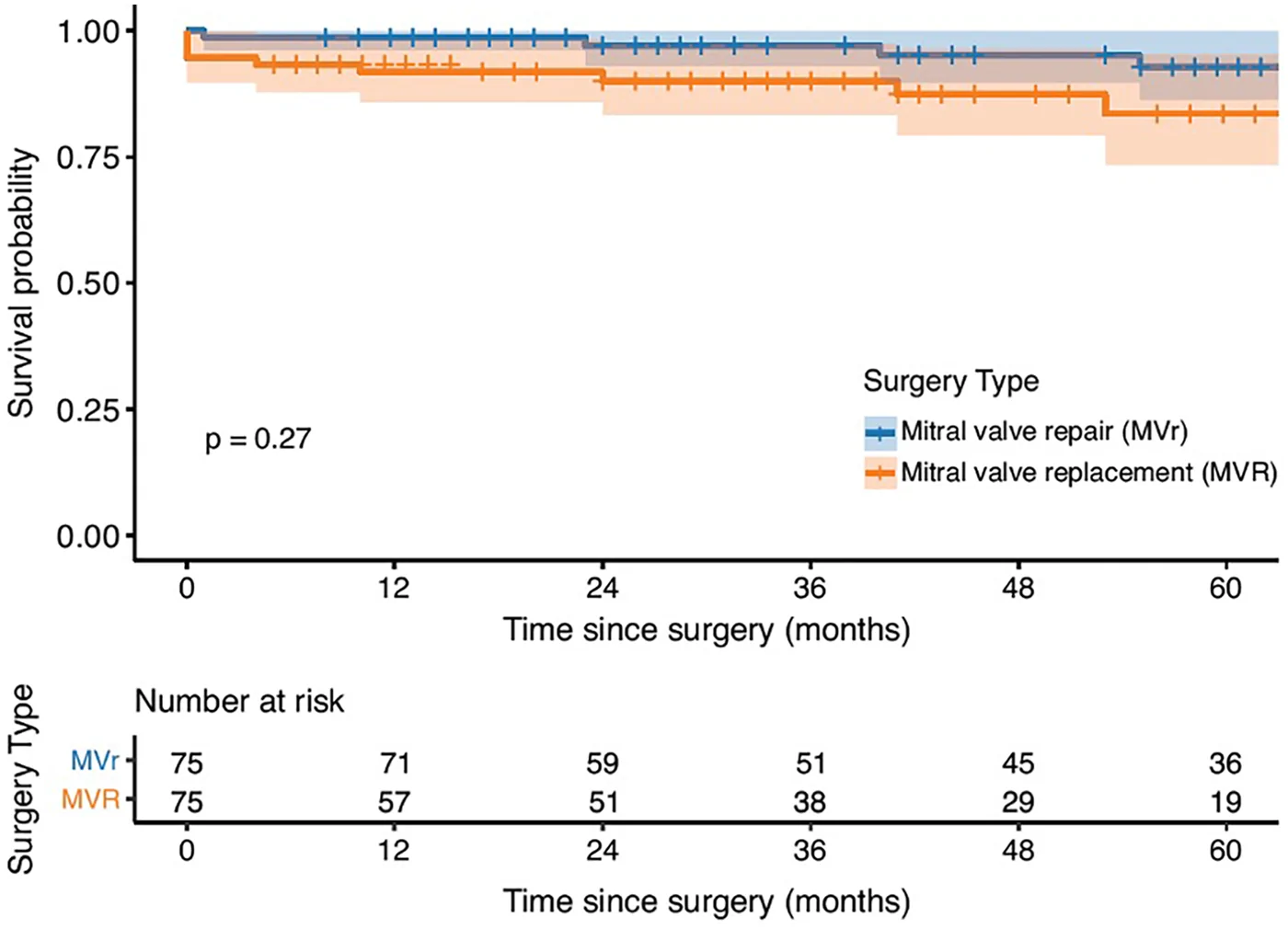
Kaplan–Meier survival curve for 5-year outcomes.

**Figure 5:**
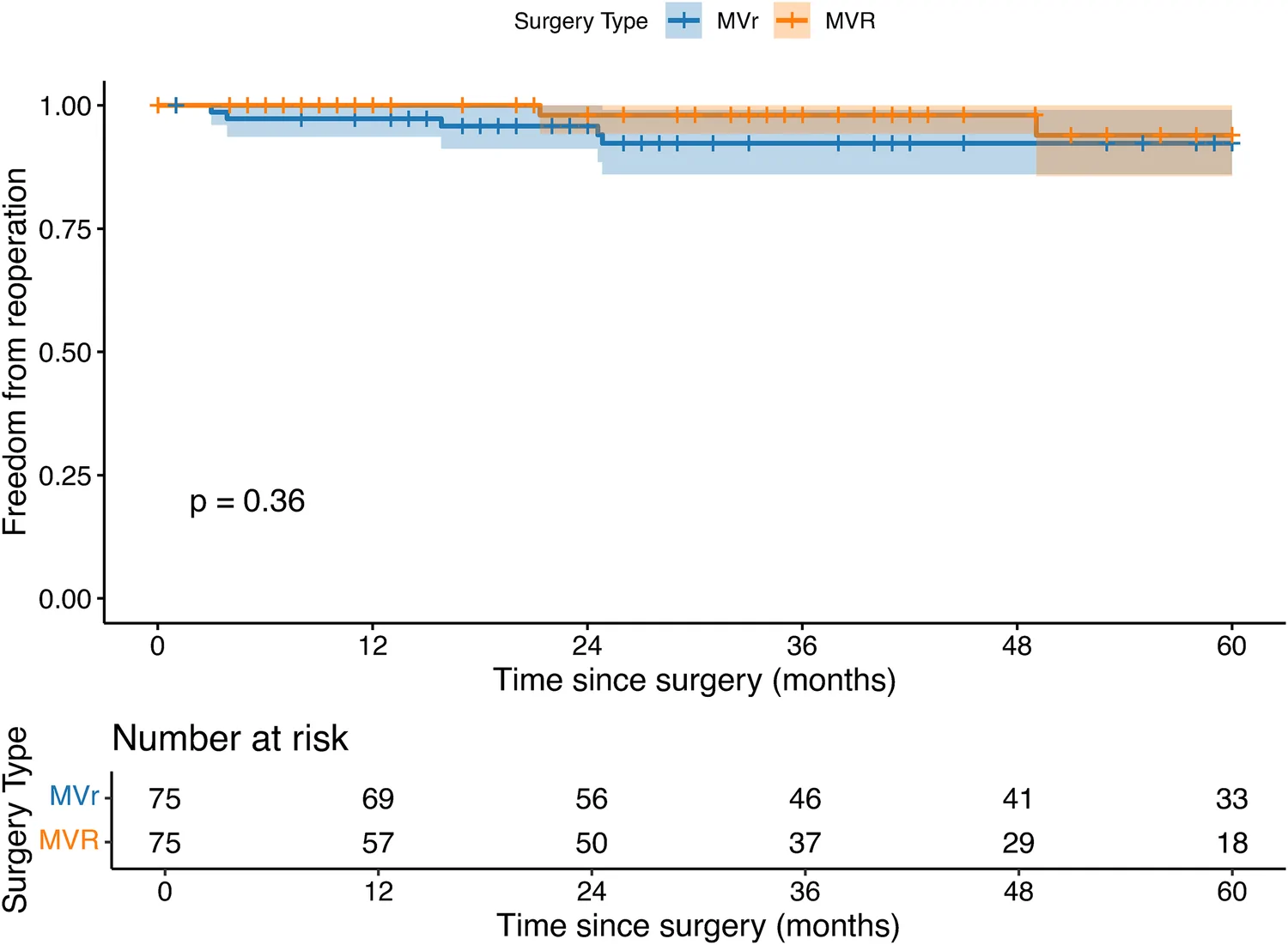
Kaplan–Meier survival curve for reoperation.

Reasons for reintervention included recurrence of significant mitral regurgitation in the MVr group (5/5), as well as prosthetic valve degeneration (1/3) and prosthetic valve endocarditis (2/3) in the MVR group. The interval between the index procedure and reintervention differed between groups: In the MVr group, reintervention occurred at a median of 16 months (range 3–25 months), with an early cluster at 3–4 months suggestive of technical or procedure-related failure and later events (16–25 months) more consistent with recurrent or progressive MR. In the MVR group, reintervention occurred later overall (median 49 months, range 21–99 months); the earliest event (21 months) was due to prosthetic valve endocarditis, whereas structural valve degeneration occurred substantially later (49 and 99 months), consistent with the expected durability of biological mitral prostheses.

Overall mortality during follow-up was 13.3% (*n* = 10) in the MVR group vs. 5.3% (*n* = 4) in the MVr group (*p* = 0.27) (Figure 4). Known causes of mortality included right heart failure, multi-organ failure, cardiogenic shock, and/or septic complications related to endocarditis (in-hospital), as well as non-cardiac causes such as COVID-19, pneumonia, neurosarcoidosis, and underlying malignant disease. Specifically, the three in-hospital deaths in the MVR group were attributable to septic shock from endocarditis (*n* = 1), multi-organ failure with ECMO-supported cardiogenic shock (*n* = 1), and hemorrhagic shock from hemothorax following emergency re-thoracotomy (*n* = 1).

To determine whether the mortality difference between groups persisted beyond the early postoperative period, a landmark survival analysis was performed excluding the three in-hospital deaths. Among 30-day/in-hospital survivors (*n* = 72 MVR, *n* = 75 MVr), 5-year survival was 87.1% in the MVR group vs. 92.9% in the MVr group, with no significant difference between groups (log-rank *p* = 0.147), indicating that the observed mortality difference is concentrated in the early postoperative phase.

### Sensitivity analysis (IPTW)

IPTW analysis on the full cohort (complete-case *N* = 692) demonstrated a significant positivity violation, with a maximum weight of 3,284 among control patients, reflecting the absence of comparable MVr candidates for the highest-risk MVR patients. Even after weight trimming, covariate balance under IPTW was inferior to the existing matched cohort for the majority of covariates (Supplementary Table S2), with the exception of the surgical risk score, which IPTW balanced more effectively than matching.

## Discussion

In this retrospective single-center analysis, totally endoscopic MVR was required infrequently in a large cohort of patients undergoing totally endoscopic MVS. Among 1,037 consecutive patients with a totally endoscopic approach, only 75 patients (7.2%) underwent valve replacement, corresponding to a repair rate of over 90% for this cohort. This aligns with prior published data and highlights that totally endoscopic MVr can be achieved in the vast majority of patients when performed at an experienced center (22–25).

The most common reasons for performing MVR were sclerotic leaflet degeneration, including rheumatic disease and/or relevant mitral stenosis, as well as acute infective endocarditis. In addition, failed MVr and prior TEER with significant residual regurgitation contributed to the need for valve replacement. These findings are consistent with previous reports describing similar indications for MVR (26–28). Especially in infective endocarditis, leaflet destruction, annular involvement, or abscess formation may necessitate MVR (29).

The increasing use of TEER has also resulted in a growing number of patients presenting for surgical treatment after failed procedures (27, 30). Clip-related leaflet damage or fibrosis may complicate surgical repair and require valve replacement (26). Although the number of patients in this subgroup was small in the present study, failed TEER is increasingly recognized not merely as one indication among many, but as an emerging paradigm likely to substantially increase the future role of surgical MV replacement, particularly at experienced minimally invasive and endoscopic centers. Patients referred for surgery after failed TEER are, by definition, a frail population with a substantial comorbidity burden; in this context, the reduced surgical trauma associated with a totally endoscopic approach may represent a particular advantage, facilitating safe surgical rescue in patients who might otherwise be considered high-risk for conventional sternotomy.

The overall rate of MVR observed at our center is comparable to prior published data on minimally invasive MVS. In large cohorts, replacement rates typically range between 5% and 15%, depending on patient selection and center experience (22–25).

Despite the higher preoperative risk profile in the MVR group, reflected by a higher STS PROM score and greater prevalence of endocarditis, early outcomes following totally endoscopic MVR were reasonable and largely similar to those of patients undergoing MVr. In-hospital mortality and major complications were low.

Postoperative transvalvular gradients were within the expected range for biological mitral prostheses (31), and no relevant paravalvular leakage was observed. Prosthesis–patient mismatch (PPM), commonly defined by an indexed effective orifice area below a validated threshold, has been associated with higher residual gradients and poorer early and late outcomes after mitral valve replacement (32). Given a median prosthesis size of 29 mm in the present cohort, the indexed effective orifice area was not routinely calculated; nevertheless, careful prosthesis sizing and close hemodynamic follow-up for potential PPM appear warranted in this population.

When compared with a propensity-matched cohort undergoing totally endoscopic MVr, intraoperative metrics such as cross-clamp, cardiopulmonary bypass, and total procedure times were similar. Conversion to sternotomy occurred more frequently in the replacement group, likely reflecting the greater complexity of the underlying valve pathology.

Patients undergoing MVR had a longer hospital stay and higher rates of permanent pacemaker implantation. The increased pacemaker rates likely reflect both the complexity of the underlying pathology and the greater potential impact of prosthesis implantation on the atrioventricular conduction system (33, 34).

Despite these differences, in-hospital mortality remained low and did not differ significantly between groups, although in-hospital deaths occurred only in the replacement cohort. During follow-up, reoperation rates were low, and overall survival in the MV replacement group remained acceptable. Mortality during follow-up was higher in the replacement group (13.3% vs. 5.3%), although this difference did not reach statistical significance. Reoperations were mainly driven by prosthetic valve endocarditis and structural valve degeneration and remained infrequent, highlighting the structural durability of predominantly implanted biological prostheses within the analyzed follow-up period. Notably, the timing pattern supports this interpretation: reinterventions after MVr occurred earlier and included a subset within the first 3–4 months, whereas reinterventions after MVR occurred later and predominantly reflected structural valve degeneration rather than early technical failure.

The systematic use of chordal-sparing techniques during MVR may also have contributed to the favorable postoperative functional results observed in our cohort. Preservation of the subvalvular apparatus has been shown to support postoperative left ventricular function and remains an important surgical principle (35–37).

These findings confirm that MV repair remains the preferred treatment strategy whenever feasible. At the same time, totally endoscopic chordal-sparing MV replacements represent a safe and effective alternative in selected patients with complex and non-repairable MV pathology, including extensive leaflet sclerosis, rheumatic disease, infective endocarditis, or prior TEER.

Thirty-day survival differed significantly between groups (94.7% vs. 100.0%, log-rank *p* = 0.043), with all early deaths occurring in the replacement cohort; permanent pacemaker implantation, hospital stay, and postoperative TAPSE also differed significantly between groups. This finding should not be interpreted as evidence of clinical equivalence, but rather reflects the substantially higher baseline surgical risk of patients requiring replacement (elevated STS PROM, higher prevalence of endocarditis) despite propensity matching. Mid-term outcomes, including reintervention rates and overall survival to last follow-up, remained comparable between groups, suggesting that totally endoscopic MVR can achieve acceptable mid-term outcomes in appropriately selected, higher-risk patients despite the early survival difference.

Several limitations should be acknowledged. First, this study represents a retrospective analysis from a single high-volume center with extensive expertise in totally endoscopic MVS, which may limit the generalizability of the findings. Second, the number of patients undergoing MV replacement was relatively small compared with the overall cohort. Third, although propensity score matching was performed to reduce baseline differences, matching was constrained to a predefined set of variables, and residual differences in variables such as STS PROM, endocarditis, hemoglobin, and mitral valve pathology persisted after matching. Given the marked imbalance in group size between the replacement and repair cohorts (75 vs. 962 in the full institutional database), an IPTW sensitivity analysis using an expanded covariate set was performed but was limited by a severe positivity violation (maximum weight 3,284) and did not materially improve covariate balance compared with the existing matched cohort (Supplementary Table S2); therefore, residual confounding related to these incompletely balanced factors cannot be excluded and should be considered when interpreting the comparative outcomes. Fourth, systematic postoperative echocardiographic data on left ventricular volumes were not available in this cohort; potential differences in left ventricular reverse remodeling, including possible volumetric mismatch, between chordal-sparing replacement and repair could therefore not be assessed and warrant dedicated prospective evaluation in future studies.

Overall, totally endoscopic mitral valve replacement was required only rarely in this large series of minimally invasive mitral procedures. When necessary, however, chordal-sparing replacement provided acceptable early and mid-term outcomes and represents a reasonable treatment option for patients with complex and non-repairable mitral valve pathology.

## Conclusion

Totally endoscopic chordal-sparing mitral valve replacement is required infrequently at high-volume centers, accounting for only 7.2% of all totally endoscopic MV procedures in this large consecutive series. The most common indications included sclerotic leaflet degeneration, infective endocarditis, and MV stenosis, alongside failed prior repair attempts and residual MR following transcatheter edge-to-edge repair—an emerging and increasingly relevant clinical scenario.

Despite a significantly higher preoperative risk profile in the replacement group, as reflected by elevated STS PROM scores and greater comorbidity burden, early and mid-term outcomes were acceptable. In-hospital mortality remained low, perioperative complication rates were largely comparable to those of matched repair patients, and no paravalvular leakage was observed. Notably, permanent pacemaker implantation rates and hospital stays were higher after replacement, reflecting the complexity of the underlying pathology. During follow-up, reintervention rates were low and similar between groups, and 5-year survival remained acceptable, with no statistically significant difference between cohorts.

The systematic application of chordal-sparing techniques likely contributed to the preservation of left ventricular function and favorable postoperative results. MVr remains the preferred surgical strategy whenever feasible. However, in patients with complex, non-repairable pathology, totally endoscopic chordal-sparing MVR performed at an experienced center represents a safe and clinically effective alternative, delivering outcomes comparable to MVr regarding reintervention and mid-term mortality.

## Data Availability

The data analyzed in this study is subject to the following licenses/restrictions: No restrictions. Requests to access these datasets should be directed to f.sobik@uke.de.
