# Supplementary material for: Totally endoscopic mitral valve replacement: reasons for non-feasibility of repair and 5-year outcomes
Source: Front Cardiovasc Med. 2026 Oct 20;13:1903773. doi: 10.3389/fcvm.2026.1903773 (PMC13727415; doi:10.3389/fcvm.2026.1903773)
Supplement: Supplementary file 1 [file Table1.docx]

**Supplementary Table 1. Baseline characteristics according to indication for mitral valve replacement**

| **Subgroup** | **n** | **Age, years** | **Male, n(%)** | **STS PROM, %** | **LVEF preop, %** | **In-hospital mortality, n(%)** | **Reoperation, n(%)** |
| --- | --- | --- | --- | --- | --- | --- | --- |
| Sclerotic leaflet degeneration | 21 | 62.3±12.0 | 11 (52.4%) | 1.82±1.21 | 57.2±7.9 | 0 (0.0%) | 0 (0.0%) |
| Acute MV endocarditis | 20 | 67.3±7.0 | 10 (50.0%) | 2.06±1.21 | 61.8±6.9 | 1 (5.0%) | 1 (5.0%) |
| Moderate/severe MV stenosis | 13 | 60.5±6.8 | 3 (23.1%) | 1.82±1.04 | 54.8±6.3 | 0 (0.0%) | 0 (0.0%) |
| Failed MV repair attempt | 13 | 65.8±8.9 | 5 (38.5%) | 2.03±1.33 | 61.2±7.5 | 1 (7.7%) | 0 (0.0%) |
| Rheumatic disease | 9 | 57.7±6.4 | 3 (33.3%) | 1.31±0.70 | 54.0±4.9 | 0 (0.0%) | 0 (0.0%) |
| Failed M-TEER | 5 | 69.0±5.3 | 2 (40.0%) | 2.82±1.70 |  |  |  |
