# Supplementary material for: Totally endoscopic mitral valve replacement: reasons for non-feasibility of repair and 5-year outcomes
Source: Front Cardiovasc Med. 2026 Oct 20;13:1903773. doi: 10.3389/fcvm.2026.1903773 (PMC13727415; doi:10.3389/fcvm.2026.1903773)
Supplement: Supplementary file 2 [file Table2.docx]

**Supplementary Table 2. Sensitivity analysis (IPTW)**

| **Covariate** | **SMD 1:1 matched cohort (N=150)** | **SMD Unadjusted full cohort (N=692)** | **SMD IPTW raw (N=692)** | **SMD IPTW trimmed@10 (N=692)** | **SMD IPTW trimmed@5 (N=692)** |
| --- | --- | --- | --- | --- | --- |
| Age, years | 0.031 | 0.374 | 2.363 | 0.343 | 0.271 |
| Male sex | -0.062 | -0.495 | 1.135 | -0.049 | -0.152 |
| BMI, kg/m² | 0.015 | 0.02 | 2.47 | 0.082 | -0.011 |
| Atrial fibrillation | 0.379 | 0.432 | -1.331 | -0.285 | -0.198 |
| Arterial hypertension | -0.24 | 0.144 | -1.331 | -0.275 | -0.188 |
| STS PROM, % | 1.218 | 1.362 | -4.433 | -0.329 | -0.104 |
| Functional pathology | -0.461 | -0.113 | -3.757 | -0.632 | -0.466 |
| Other pathology | 0.21 | 0.464 | 0.585 | 0.354 | 0.331 |
| LVEF preop, % | 0.177 | -0.091 | 3.648 | 0.298 | 0.155 |
| NYHA class | 0.316 | 0.572 | 1.059 | 0.255 | 0.211 |
| TAPSE, mm | -0.09 | -0.39 | 1.535 | 0.368 | 0.283 |
